# The organelle of differentiation in embryos: the cell state splitter

**DOI:** 10.1186/s12976-016-0037-2

**Published:** 2016-03-10

**Authors:** Natalie K. Gordon, Richard Gordon

**Affiliations:** Retired, University of Manitoba, Winnipeg, Canada; Embryogenesis Center, Gulf Specimen Aquarium & Marine Laboratory, 222 Clark Drive, Panacea, FL 32346 USA; C.S. Mott Center for Human Growth & Development, Department of Obstetrics & Gynecology, Wayne State University, 275 E. Hancock, Detroit, MI 48201 USA

**Keywords:** Cell state splitter, Cell organelle, Cytoskeleton, Differentiation, Embryo

## Abstract

The cell state splitter is a membraneless organelle at the apical end of each epithelial cell in a developing embryo. It consists of a microfilament ring and an intermediate filament ring subtending a microtubule mat. The microtubules and microfilament ring are in mechanical opposition as in a tensegrity structure. The cell state splitter is bistable, perturbations causing it to contract or expand radially. The intermediate filament ring provides metastability against small perturbations. Once this snap-through organelle is triggered, it initiates signal transduction to the nucleus, which changes gene expression in one of two readied manners, causing its cell to undergo a step of determination and subsequent differentiation. The cell state splitter also triggers the cell state splitters of adjacent cells to respond, resulting in a differentiation wave. Embryogenesis may be represented then as a bifurcating differentiation tree, each edge representing one cell type. In combination with the differentiation waves they propagate, cell state splitters explain the spatiotemporal course of differentiation in the developing embryo. This review is excerpted from and elaborates on “*Embryogenesis Explained”* (World Scientific Publishing, Singapore, 2016).

“Circuit Diagram for a Sea Urchin…. From a Thompsonian perspective [1], this thread of work is incomplete in one crucial way. Although it provides a way of thinking about the control of genes, the work to date, to my knowledge, does not make the link to form. We still lack the simulator that can take the… diagram [Fig. 1] and compute a video of a developing sea-urchin embryo as output. That is no criticism of the work of Davidson and colleagues [2], who have taken us a huge step in that direction. Just an observation that there is a ways yet to go” [3] (citations and updated figure added).

**Fig. 1:**
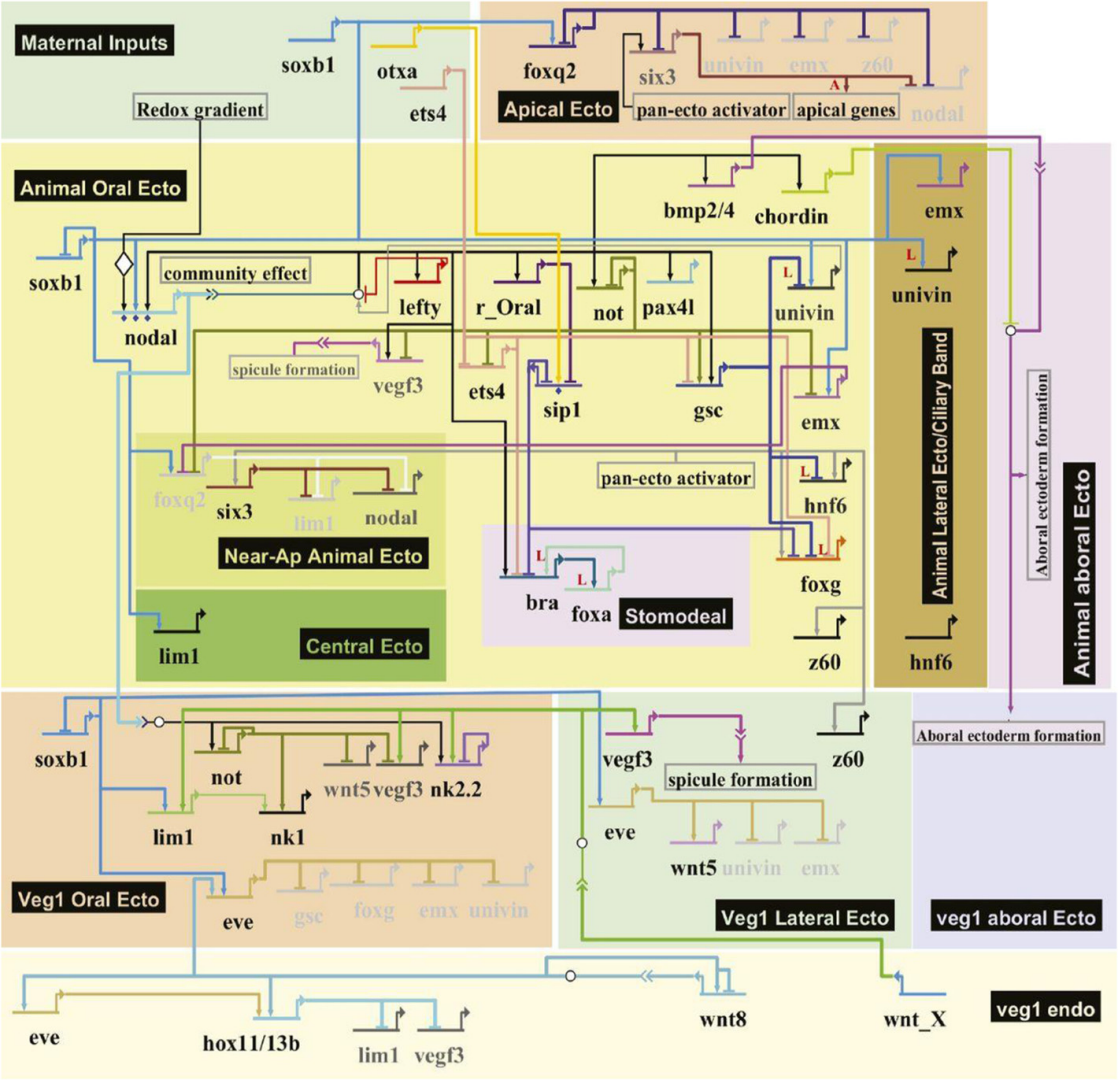
“The GRN [Gene Regulatory Network] model illustrating the genomic control of 2D expression pattern formation in the sea urchin ectoderm. This model is a BioTapestry presentation of all interactions among regulatory genes governing ectoderm regulatory state diversification up to the onset of gastrulation. The circuits show that domain-specific repressors are commonly used to define the boundaries along both embryonic axes.” From [2] with blanket permission for use “in a review article” per http://www.pnas.org.uml.idm.oclc.org/site/aboutpnas/rightperm.xhtml

## Background

Multicellular organisms are made of 4D spatiotemporal arrays of different cell types. The identity and function of any individual cell is defined and presumably determined by the specific complement of proteins and RNA that the cell contains. Proteins and RNA are encoded in the genome. While each cell has a complete copy of the whole genome, each cell uses only a portion of the total gene products encoded in it. The mechanism by which this process of differentiation of the one-cell embryo (i.e., the fertilized egg) into more than 3.72×10^13^ cells [4] of up to 7000 cell types [5, 6] is accomplished over space and time is the central puzzle of embryology. Here we will show how the gene regulatory networks [2, 7] of the late Eric H. Davidson [8] (Fig. 1) are linked to embryo form over time, i.e., to embryogenesis. The key to this is our prediction [9] and discovery [10, 11] of the organelle of differentiation, a cytoskeletal apparatus we call the cell state splitter, that propagates differentiation waves from cell to cell as it initiates a step of differentiation within its cell. This review is excerpted from and elaborates on a book length exposition [12] (with permission of World Scientific Publishing). Our work grows out of our research on the embryogenesis of urodele amphibians, especially the newt *Taricha torosa* [13] and a neotenic salamander, the axolotl (*Ambystoma mexicanum*) [14].

There is a widespread assumption that the environment somehow determines which cells in an embryo become which kinds. At every step of development, each cell is presumed to react to its local environment within the embryo in order to “decide” via the genes what to do next, and this concept of cells and genes making decisions permeates much thinking about embryogenesis. The major problem with such thinking is that each cell contains the same DNA, and passes identical copies on to its daughter cells (barring mutation and a few rare exceptions), so that there is no obvious reason or genetic mechanism for cells to create new environments for one another. That all of these interactions between genetically identical cells should somehow work themselves out in the creation of many distinct microenvironments, all in the right place at the right time, is about as plausible as having a musically untrained crowd of chattering people suddenly switch their cacophony to four part harmony and perform Mozart’s complete *Ave Verum Corpus* [15]. Symmetry breaking considerations may help for one step, but lead to a combinatorial nightmare for differentiation [16], the process by which multiple cell types are generated from the single fertilized egg cell in a multistep, branching process. Thus notions of environmental control of development have to be reexamined. As one author put it “…the embryo is provided with little more spatial information than the simple instruction: ‘This side up’” [17]. Given that inverted embryos [18] or those in zero gravity [19] can develop pretty normally, even “this side up” may not be “provided”. Localized, so-called “maternal determinants” are neither universal nor determining, as seen in experiments with inverted embryos that redistribute those “determinants” but nevertheless develop normally in many cases [18]. We will attempt to resolve this ancient issue here, in a physics context.

Early embryogenesis generates ectoderm, mesoderm and endoderm, which represent the three precursor cell types from which all future cell types are derived. All types of tissue in the adult organism can be traced back to one of these three tissue types, a process called fate mapping. Fate maps are similar for most animals [20]. These three early cell types are commonly called the “germ cell” layers [21]. Ectoderm gives rise to the skin, brain, spinal cord and nerves. Mesoderm eventually forms all the middle parts of the embryo such as the muscles [22] and skeleton [23, 24]. Endoderm forms all future interior parts such as liver [25], pancreas and intestines. The heart, like other important organs in the gut and chest, has an outer layer of mesodermal origin and an inner layer of endodermal origin [26, 27].

The same determination of three essential germ cell layers occurs in birds, fish and mammals. Mammals have that extra set of cells (trophoblast cells), the ones which form the placenta and support tissue but as those are discarded at birth their formation is not counted as a germ cell layer. In birds the embryo forms as a flattened plate with the three cell types on top of the undivided yolk. In zebrafish (*Danio rerio*) the three germ layers are similar with a more rounded embryo but the same basic layout.

In mammals, the three layers are formed from the inner cell mass (inside the trophoblast) as the embryo develops. In fact, this common body plan goes all the way back to our first vertebrate ancestral forms. Invertebrates also have the same essential three layers pushing the origin of ectoderm, mesoderm and endoderm even further back along our common ancestral tree. Sea urchins have a fate map remarkably similar to amphibians [20]. Insect fate maps include mesoderm, endoderm, and the ectoderm equivalent known as dorsal epidermis.

Once the three germ layers are determined, the embryo begins two of the most marvelous and complicated stages of development imaginable. These stages are called gastrulation and neurulation. This simple three-layered embryo changes itself to form the basic body plan of the adult organism. By the end of gastrulation the embryo will be a complex multilayered organism with a front end and a back end and a clearly defined top (dorsal side) and bottom (ventral side), with future internal organs ready to be created. By the end of neurulation in vertebrates, a future spinal cord and brain are set in place and the whole outside is also completely covered with a layer of future skin tissue.

**Fig. 2:**
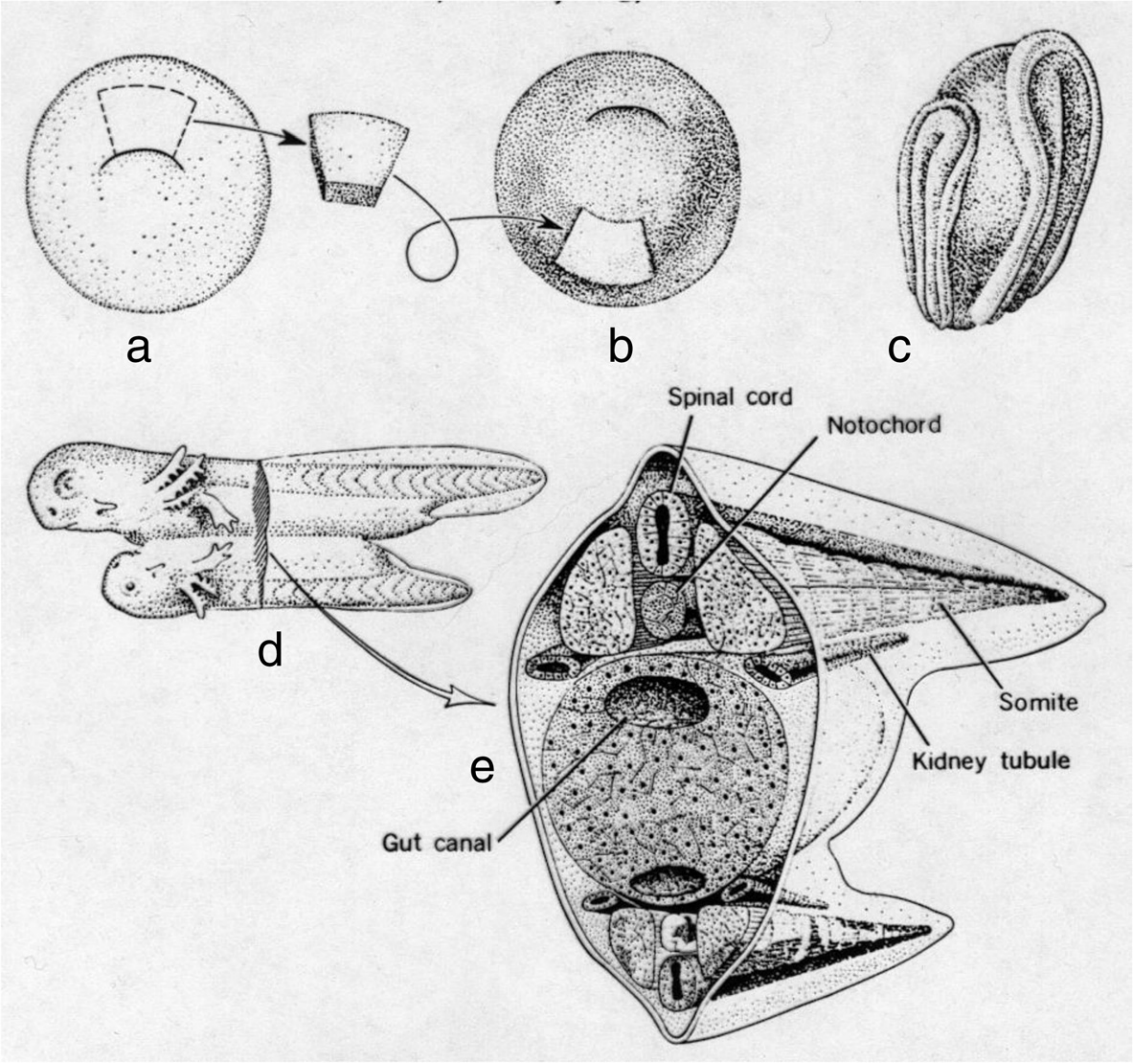
Transplantation of a piece of ectoderm containing a portion of the dorsal lip of the blastopore from lightly pigmented embryo **a** to darkly pigmented embryo **b** results in a second neural plate (**c**). The result is a double embryo, i.e., conjoined twins (**d**, **e**). This shows that any portion of the ectoderm is capable of producing a neural plate and subsequent development. In an ordinary, single embryo the dorsal (top) part of the ectoderm produces the brain and spinal cord, while the lower (ventral) hemisphere produces the epidermis, which becomes skin. This sketch, which depicts the original experiments by Hilde Mangold and Hans Spemann [30], is by Victor Twitty. From [182] with permission from Macmillan Education

The most famous experiment in embryology is the discovery of neural induction, for which Hans Spemann won the Nobel Prize in 1935 [28, 29]. The basic experiment, performed by his graduate student Hilde Mangold in 1921 and 1922 [30, 31], is illustrated in Fig. 2. The dorsal lip of the blastopore is an arc of cells at the bottom of an amphibian embryo. Mangold cut this out and transferred it to a hole cut in the ventral ectoderm in another embryo. To distinguish the transplant, which healed in, from the recipient embryo, two different species of the newt *Triton* were used, whose eggs have different surface concentrations of pigment granules. The remarkable result was the appearance of a second neural plate. The donor organizer somehow “induced” the surrounding ectoderm of the host to become a new neural plate, which went on to become a conjoined twin. The transplanted piece of tissue was called an “organizer” because most of the tissue in the new neural plate came from the host and was thus was “organized” by the small piece that was patched in to become a second embryo.

The problem then took a peculiar turn. Embryologists reasoned that neural induction could be physical, or it could be chemical. If it’s physical, it’s too hard to understand. Therefore it is probably chemical. This illogical reasoning led to an unending search for “the morphogen”, *the* chemical that came out of the organizer and induced the secondary neural plate (§2.04 in [5]). No further thought was given to a physical explanation.

Given the discovery of physiological gradients in embryos [32, 33], it then became common for embryologists and molecular biologists to speak of a “morphogen gradient” across developing tissue that begins at the site of neural induction, creating a gradient of gene products or “morphogens” across the ectoderm. Morphogens are the basis for the concept of positional information [34] which presumes that a cell can know its position by “reading” the concentration of the molecules of the gradients and then deciding what it is supposed to do, by “looking up” its coordinates in some sort of stored table in its DNA. With these epicycles the problem of embryogenesis was “solved” and, like the far more quantitative Ptolemaic version of the solar system [35, 36], permeated textbooks and teaching for an extended period of the history of science. Morphogens are still widely taught, along with gene regulatory networks, as a full explanation of embryogenesis.

There are numerous problems with the morphogen gradient model:

1. In order to maintain a gradient at steady state, besides a steady [37], spatially defined [38] source for the diffusing molecules, there has to be a sink. This means there must be a way in which diffusing molecules are destroyed or removed along the way and/or at some boundaries [39]. Most people who invoke gradients don’t bother with analyzing and solving the partial differential equations for diffusion of molecules to show how the gradients work. Sinks are rarely, if ever, even considered when the gradient model is invoked.
2. A common supposition is that the molecules diffuse outside cells instead of through them [40]. This is a convenient assumption, because it permits one to ignore the cellularized structure of space inside an embryo, But even so diffusion must occur in a confined space, if a gradient is to be established. Otherwise the molecules will just diffuse away. If the diffusion is extracellular, then its course is critically dependent on the existence of such confining boundaries. Most of the early development of the axolotl occurs in the outside layer of cells, and is normal whether or not the jelly layers and vitelline membrane are present. So in this case no such confined space exists.
3. The speed of development may not permit steady state to be reached [41–44]. This is sometimes considered an advantage in cases where the steady state could not possibly lead to the correct morphology. So we have a steady state invoked except when we don’t want it [45, 46]. In any case, the rate of development varies substantially with temperature over a species’ temperature range for normal development. This would have to be matched to the temperature dependence of diffusion of the molecule [47] which is itself dependent on the temperature variation of the viscosity of the medium through which the molecule diffuses [48].
4. Ordinary diffusion gradients do not scale well [49, 50]. The consequence for embryos is that for embryos of different sizes there should be widely different proportions of parts but we know that is not the case [51]. There is a limit on the “range” of a morphogen gradient [52]. Such range limits also limit their potential role in growing tissues [53, 54]. Amphibian embryo eggs vary from 0.75 mm to 35 mm [55], and yet produce adults with substantially the same body plan. As we have a common ancestor with amphibians, our own eggs at 0.07 mm extend the linear size range down by another order of magnitude.
5. Diffusion gradients follow the superposition principle. This means that a gradient of one substance in, say the x-direction, and a gradient of the same substance in the y-direction, result in a single one-dimensional gradient in the diagonal direction, not a two dimensional gradient. Yet biologists frequently invoke a two dimensional gradient to get the gradient model to fit their data. If you want a two dimensional gradient system you have to have two morphogen gradients with two different sources and sinks placed approximately perpendicular to one another, and three to invoke the third spatial dimension. That’s 6 unidentified sources and sinks.
6. The fundamental principal of gradients is that cells in high concentrations will respond in one way, while those at low concentrations respond in a different way while those in the middle respond in yet another way. Fluctuations in gradients always occur, especially if the number of diffusing molecules is low. Fluctuations of purported morphogen concentrations make response to particular concentration thresholds problematic [56–58].
7. Each cell has to be able to “read” the morphogen concentration accurately [59–64], lest boundaries between tissues become ragged [65]. Gradients are frequently invoked without any explanation how a cell measures a concentration. Yet in embryos boundaries between tissues are generally sharp, at the cellular level [66, 67].

Many doubts about the functioning [68, 69] or existence [70] of these so-called “morphogen” gradients have been raised, with alternatives [71, 72] and elaborations [73–82], and transport mechanisms other than diffusion [83–86] being proposed. Like epicycles, multiple, overlapping gradients in the same direction are sometimes required [87]. We won’t review the numerous molecules that have been proposed to be morphogens, but as new biologically active molecules are discovered, they tend to be added to the list [88] and then later sometimes removed [89]. While gradients such as bicoid in *Drosophila* one-cell embryos are indeed found [90], the existence of polyembryonic wasps reasonably similar to *Drosophila* in adult appearance, whose fertilized eggs split into up to 2000 embryos [91], raises doubts as to the importance of even these intracellular maternal gradients for embryogenesis.

We have another explanation for differentiation which provides the missing three dimensional and time information the embryo works with to build itself from a fertilized egg. We will begin explaining by summarizing what we know about the functioning of the DNA.

## The role of gene regulatory networks in embryogenesis

In eukaryotes, we make a broad classification of all genes in a given cell type into *exposed* and *sequestered*, a terminology first proposed by Theodore T. Puck [92–94]. Exposed genes are available for transcription. Exposed genes may or may not be actively transcribed. They are available to be transcribed if required. Sequestered genes are not available to be transcribed. We assume there are at least six levels of sequestration of DNA. DNA at any of these levels of sequestration cannot be transcribed. All 6 levels must be unraveled for the DNA to be exposed. Unraveling sequestered DNA is commonly called decondensing, usually without specification of the level of sequestration of the DNA under consideration. In the current state of the art, we can identify the following six states of sequestration, adding the last DNA level which is the single state of exposure:

1. *Mitotic sequestration*: the whole genome is condensed in preparation for mitosis. This includes crosslinking of sister chromatids by cohesins. There is little to no gene expression that can occur.
2. *Single chromosome sequestration*: a whole chromosome remains sequestered by cohesins after mitosis. X inactivation in mammals is an example where this level persists throughout the cell cycle for the X chromosome. (A few genes may remain exposed.) This kind of sequestration may include a layer of proteins that covers the chromosome.
3. *Heterochromatin sequestration*: regions of each chromosome are sequestered with clustered loop-linked condensins. Other regions, referred to as euchromatin, are not. Heterochromatin has been classified into two categories. Constitutive heterochromatin consists of stretches of DNA that contain no genes, but may have structural functions, such as telomeres and centromeres. Whether this is protein coated in any way or produces noncoding RNA is unknown. Facultative heterochromatin contains genes that are sequestered by deacetylated histones and/or noncoding RNA.
4. *Compacted nucleosome sequestration*: facultative heterochromatin whose histones are at least partially acetylated and are not coated with noncoding RNA/protein complexes nonetheless remain sequestered. The 3D arrangement of the nucleosomes at this level is currently under dispute.
5. *Nucleosome sequestration*: nucleosomes are not compacted against each other, but the DNA is still tightly wound around the nucleosome, and cannot express its genes.
6. *Nucleosome unbound sequestration*: a segment of DNA is separated from nucleosomes but nevertheless sequestered due to bound protein and/or RNAs. Two mechanisms have been proposed, both of which may be used: the nucleosomes slide along the DNA out of the way, or the DNA loops out from the histone octomer it is attached to. The latter is known as the “seatbelt model” because you can visualize it by imagining pulling the shoulder strap of your seatbelt away from your chest.
7. *Exposed*: a segment of DNA is available for transcription. This may require removal of repressors and the formation of a regulatory archipelago (defined below).

One of the key kinds of proteins involved in controlling gene sequestration is the transcription factor. The initial concept of a transcription factor was actually rather muddy. It began by scientists finding a protein in a cell. The appearance of the protein, a change to the gene that coded for the protein, or removal of the protein altogether by blocking it, were associated with altered levels of proteins coded for by other genes. The alteration of protein expression could be either up or down. Any protein that was associated with changes in gene transcription levels of another protein was then labeled a transcription factor. Today, a transcription factor is more precisely defined as any protein that binds (or is part of a complex that binds) to a specific sequence of DNA and by that binding, changes the rate of transcription of a gene. For our purposes a transcription factor can act to sequester or expose a gene at any of the above levels.

RNA polymerase, contrary to how it is often pictured, is not floating about randomly in the nucleus looking for an exposed gene to jump on and transcribe. Rather RNA polymerase is part of a complex of proteins that forms the transcriptional machinery. The machinery is anchored to the nucleoskeleton in areas separate from chromosome territories. Decondensed loops of DNA are also in this area, moved in by the same enzymatic protein machinery that decondenses and thereby spreads the loop. Only exposed DNA can be accessed by the RNA polymerase and transcribed.

**Fig. 3:**
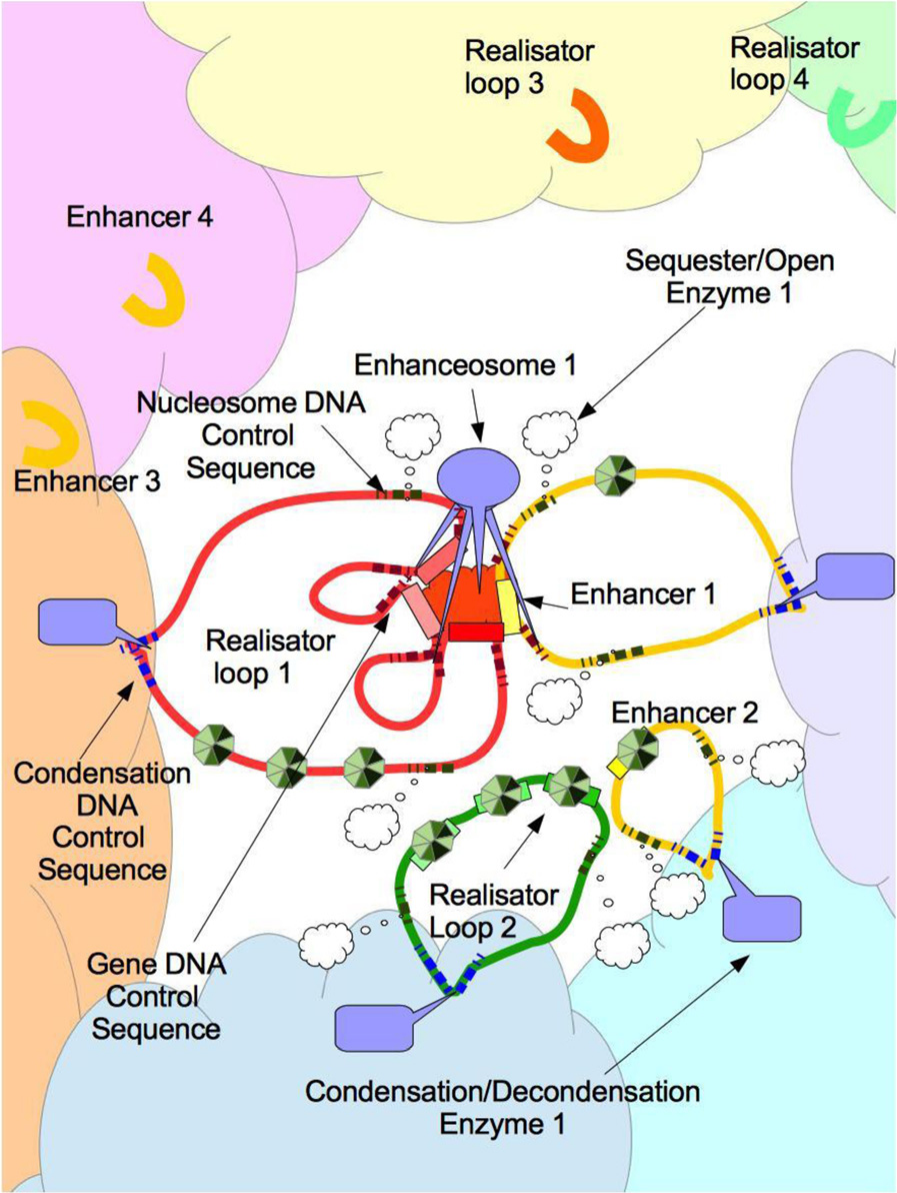
The nucleus has areas of chromosome territories (colored clouds) and regions that are open containing RNA polymerases (central orange cloud). A specific set of transcription figures creates an enhanceosome which binds to a specific enhancer. It also binds to a realisator gene which is on its own loop divided into introns and exons. If the gene and enhancer is open, as in realisator loop 1, the DNA can be bound to the enhanceosome if the correct transcription factors are present and form a regulatory archipelago. The gene is then transcribed. If the DNA is sequestered, as in Realisator Loops 2, the DNA cannot be bound the enhanceosome even if the correct transcription factors are present. If the DNA is condensed (as in Realisator Loops 3 and 4 and their enhancers) it completely inaccessible

The attachment points of exposed DNA can create loops that bring together parts of a gene or more than one gene into juxtaposition with the RNA polymerase complex for transcription. This collection of the smallest organizational loops of DNA which are bound to the polymerase and held by the enhanceosome complex of transcription factors is called a “regulatory archipelago” (Fig. 3). The stretch of DNA that is noncoding between the enhancer DNA sequence and the gene that it enhances is a “gene desert”. Like most deserts, we are finding there is actually a lot of regulatory life in there even if it isn’t in standard gene form.

Within each gene desert, DNA in the genome also contains many specific kinds of enhancer binding sites. Like the term ‘transcription factor’, ‘enhancer sequence’ began as a rather nondescript term. If you changed the sequence of the DNA and decreased transcription or you found a transcription factor that could bind to that sequence and in doing so cause an increase in transcription, then that DNA sequence is an enhancer. There are literally tens of thousands of these DNA enhancer regions that can potentially attract transcription factors. Enhancers are spread all over the genome in a nonrandom fashion. About one third of enhancers are located near the promoter region of the gene they enhance, as would be expected. However 2/3 of enhancers are located on entirely different regions of the DNA. There are even enhancers from one chromosome that can affect transcription of a gene on another chromosome.

We now know that several loops of DNA from different parts of the genome can move out of the sequestered chromosome territories, become exposed and move into an area where RNA polymerase complexes are functioning. A transcription factor can bind both an exposed enhancer region on one loop of DNA and the gene promoter on another loop containing the gene. Both loops move to the transcription machinery where the gene is transcribed. And so enhancers that appear to be far away from the gene they control in the linear DNA sequence can be brought close together by the archipelago looping structures of the genome. Splice variants of genes can also be made by changing the loops used in the regulatory archipelago (Fig. 3). And so the three dimensional state of exposed DNA as well as the enhancer regions becomes important for gene expression. The 3D structure depends on the transcription factors that are bound to the DNA.

In addition to the enhancer regions and looping sites in exposed DNA, there are other sequences on sequestered DNA and higher ordered looping structures. DNA that is sequestered will have specific patterns of histone modification that are used in further higher order organization. Loop structure is also hierarchical in nature. At the higher levels of sequestration, proteins interact and bind with lower order organizational signals based on proteins bound to histones and specific DNA sequences. Just as in exposed DNA, the three dimensional structure of higher order sequestration of DNA is also encoded in the linear DNA sequence.

Some transcription factors are quite general and found in all cell types. These are “housekeeping” transcription factors. Typically 50 % of the DNA in a differentiated cell is exposed. Housekeeping genes exist in all organisms. (Some scientists prefer the term “reference genes” for which there is an official representative list [95].) These are genes that all cells require for normal functioning, such as genes for transfer RNA. These housekeeping genes are expressed constitutively in all tested cell types at all stages of life and thus always fall into the exposed category. Humans have 3804 known housekeeping genes [96] representing about 15-19 % of all 20,000 to 25,000 genes [97]. There may be many more housekeeping proteins than the 3804 genes since housekeeping genes, like tissue specific genes often have multiple splice variants.

If a transcription factor affecting a gene is exposed and conditions are right, the gene will be transcribed and then translated. If the DNA is sequestered, the gene won’t be affected because the transcription factor can’t bind to it. Each cell type will also have some transcription factors that will be found only in related tissues such as in all mesoderm derived cells. Other transcription factors will be tissue type specific, appearing in only a single tissue and only after that tissue is determined. It is this combination of common (housekeeping), less common and specific transcription factors that result in a unique set of transcriptions factors for each cell type. Each cell type will also have its own unique compliment of exposed and sequestered enhancer regions and genes.

Noncoding RNA can also bind to both DNA and transcription factors and affect transcription rates [98]. We are really only just beginning to appreciate the importance of noncoding RNA. In fact it may be that these noncoding RNAs, paired with transcription factors, are of critical importance to all steps of development working by guiding transcription factors to their places [99]. This would dramatically increase the context specific binding of transcription factors that are otherwise nonspecific.

Chromatin remodelers work at higher levels of sequestration because they regulate transcription through epigenetic alterations to the chromatin thereby changing its higher order structure rather than by binding directly to specific DNA sequences. Activity of chromatin remodelers can result in enhancer regions that were exposed, becoming sequestered and vice versa. For example, the gene for *engrailed* encodes a transcription factor which binds to the engrailed DNA domain. When bound, the Engrailed protein interacts with chromatin modeling enzymes to change sequestered DNA into exposed DNA during development. And so chromatin remodelers also regulate when genes are sequestered and when genes express but they require specific cues not directly encoded in the DNA to guide their activity.

Housekeeping genes have some fundamental differences when compared to tissue specific genes. They are highly conserved, meaning they have changed very little over evolutionary time and vary little between organisms [100]. The housekeeping genes are probably less compact [101], in the sense of containing more noncoding DNA (such as total intron length) than tissue specific genes. Housekeeping gene proteins tend to physically interact [102] with more other proteins than tissue specific proteins [103]. Most important they are almost all turned on and off with “CpG-dependent core promoters” [104]. The tissue specific genes use mostly alternative promoters, with some exceptions [101]. Some of these exceptions act just outside a “CpG island” at binding sites referred to as “CpG island shores” [105, 106].

As an example of how the understanding of genes changes over time, the idea that housekeeping genes are “less compact” is not accepted by all scientists. The less compact idea is at odds with the notion of evolutionary selection for the efficiency of translation [107] of housekeeping genes [108]. The conundrum may be due to weak statistical correlations and a nonlinear relationship between gene length and expression level [109] or selection for independent measures of gene properties [110]. The noncoding spaces might also include critical noncoding RNA still waiting to be discovered. In any case, housekeeping genes are always exposed genes. And so the genes can be thought of as one set regulated by CpG-dependent core promoters which are always exposed and another set which can be either exposed or sequestered.

We will define a *differon* here as all of the exposed genes in a given cell type. The term “differon” is not a new term [111]. The complementary set of sequestered genes we will call its *differoff*. Note that the differon includes genes that may or may not be actively transcribed at a given time.

A cell’s *transcriptome* [112, 113] is the subset of DNA in a differon that is being transcribed in a given cell at a given time. The transcriptome is all the gene pre-mRNAs and spliced mRNAs, as well as all the other regulatory noncoding RNAs that may or may not be produced as part of a gene, and the untranslated RNAs such as tRNAs and ribosomal RNAs. A cell’s transcriptome can be thought of as its phenotype, and thus a measure of the relationship between phenotype and genotype. However, there is no reason to assume that, for instance, every difference in cell shape or other parameters necessarily means a difference in a cell’s transcriptome. As an amoeba cell crawls and changes shape it may not be changing its transcriptome. As diatom shells decrease in size and shell pattern from one generation to the next [114–116], they might not be changing their transcriptomes.

The *proteome* is the set of all proteins being expressed in a given cell at a given time [117, 118]. In terms of set theory [119], then, for cell type *i*:

$$ proteom{e}_i\subseteq transcriptom{e}_i\subseteq differo{n}_i\subseteq active\_ genome\subseteq total\_DNA $$

with:

$$ differo{n}_i\cup differo f{f}_i= active\_ genome $$

and

$$ differo{n}_i\cap differo f{f}_i= empty\  set $$

where the symbol ⊆ means “is a subset of”, ∪ means “union of”, and ∩ means “intersection of”. By “active_genome” we mean the “total_DNA” except for DNA that is never used to produce anything in any kind of cell in the organism. Whether the latter is junk DNA or has structural or other functions we just haven’t learned about yet remains an open question. The housekeeping genes for a given organism with *n* cell types can be expressed in set theory as:

$$ housekeeping\_ genes={\displaystyle \underset{i=1}{\overset{n}{\cap }} proteom{e}_i} $$

**Fig. 4:**
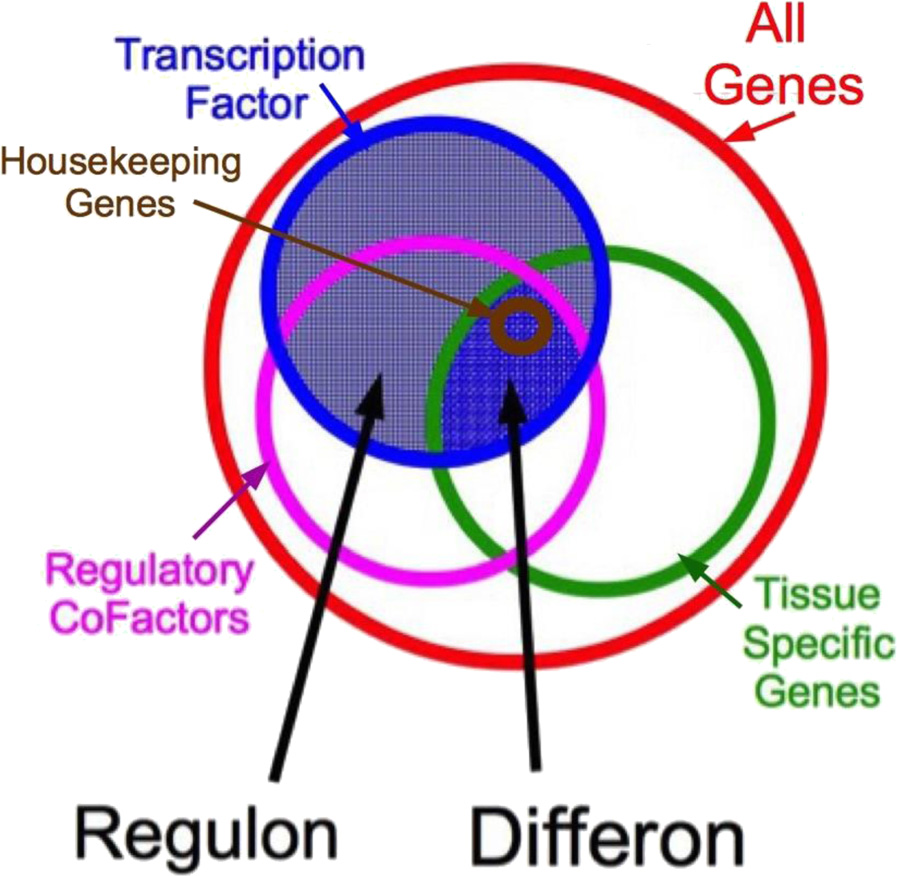
Each cell has the same set of all genes encoded in the DNA (Red circle). All cells in an organism express a set of “Housekeeping” or “Reference” genes. These genes represent certain elements such as ribosomes and components of the DNA polymerases as well as some of the cytoskeletal elements (Brown circle). There are also genes for specific transcription factors that are required for turning off and on gene expression (Blue circle hatched). Transcriptions factor gene products (i.e., proteins that are transcription factors) may be expressed in many different cell types. Many transcriptions factors require the presence of specific regulatory cofactors (Pink circle) such as small RNAs to perform some (or even all) of their functions. Expression of specific transcription factors and their regulatory cofactors often changes during development and thereby changes function of tissue specific genes. Each cell also has a set of genes that are generally specific to the tissue type that the cell belongs to but which may also be expressed in other cell types (Green circle). A Regulon is the group of a single transcription factor and all of its regulatory cofactors required to regulate a specific gene or set of genes. A regulon is defined by the genes affected by it and the regulatory cofactors associated with it and can be different in various cell types or when studied from different perspectives. A given regulon may also be functional in more than one cell type. A Differon is the set of all genes that can be expressed in a specific cell and the differon defines the cell type. All the cells with the same differon are of the same type. (The differon differs from the transcriptome, the set of all mRNA expressed in a cell, because not all genes that can be expressed are always being expressed in a cell. For example, the liver may express certain genes only in response to the presence of specific toxins and so those genes will not appear in the transcriptome if the toxin is absent but those genes are still part of the differon)

We also use the term *regulon*. In bacteria a set of genes all controlled together is known as an operon. A similar set of genes regulated by a given transcription factor, or combination of transcription factors, triggered by a discrete signal transduction pathway, is called a regulon. A regulon may be thought of as a group of genes that all work together, and are controlled in concert, with a greater complexity of interactions than that found in an operon (Fig. 4). Regulons can have significant overlaps in which genes they include. Regulons in bacteria are defined with respect to the signal that is changed and the transcription factor (usually only one) that responds to the signal. Regulon becomes a rather nonspecific term in eukaryotes with their complex gene regulation because it must be defined with respect to something. If we define a regulon with respect to transcription factors we will get certain sets of genes for each transcription factor. Since eukaryotic transcriptions factors often function differently when working in combinations with other transcription factors, eukaryotic transcription factor based regulons can have multiple, overlapping and even contradictory effects on a specific gene. If we define a eukaryotic regulon with respect to a signal like a heat shock, many transcription factors will be involved and so the set of genes could be considerably larger. Nonetheless, we would like to extend Puck’s ideas [92] to regulons by suggesting that in a given cell type, one subset of regulons is *exposed*, while the remaining regulons are *sequestered*. In this scheme, housekeeping regulons would always be exposed. In terms of set theory:

$$ differo{n}_i={\displaystyle \underset{r=1}{\overset{R_i}{\cup }} exposed\_ regulo{n}_{ir}} $$

$$ differo{n}_i={\displaystyle \underset{r=1}{\overset{R_i}{\cup }} sequestered\_ regulo{n}_{ir}} $$

*Determination* is the process by which a cell changes from having one set of exposed/sequestered regulons to a different set of exposed/sequestered regulons. While some regulons may be available to more than one tissue type, the total set of exposed regulons is unique for each cell type. It is the subset of exposed regulons that defines the cell type.

We know that there is a hierarchy of genes. We know that some genes are able to regulate and control many other genes. In genetics such genes are referred to as “master genes”. If a specific master gene (like *engrailed*) is turned on, it then activates all the genes below it in the hierarchy and at the same time can shut down others below it. If we imagine the genome is organized into multiple subsets, each subset controlled by a master gene, or key master genes, organized into regulons and differons, then a possible explanation of higher order organization becomes clearer.

Epigenetics is a higher order form of regulon. Epigenetics is the collection of all the various mechanisms, including methylation patterns on DNA and histones, which can cause some portions of the genome to be expressed and other portions to not be expressed during the life of an organism in all cells or in specific cell types. This information is added without changing the actual sequence of the DNA itself. Some of this epigenetic information for regulation of gene expression can even be inherited beyond simple cell division and can be passed from one generation to the next. For example we now know that if either your grandmother or your mother smoked during pregnancy, nicotine induced deleterious epigenetic changes can cause you to have asthma [120]. In 2008, the definition of epigenetic trait was defined as a “stably heritable phenotype resulting from changes in a chromosome without alterations in the DNA sequence” [121]. This definition is not universally accepted but it is the definition used here. Epigenetics, in our system, is primarily used for higher levels of sequestration. Even so the epigenetic markers on histones, which control higher levels of sequestration, are nonetheless added based on sequences in the DNA at lower levels of sequestration.

We imagine then, a system where the DNA is loose and exposed if it is being used, and somewhat loose but sequestered if it is not being used now, but might be required soon. The DNA of genes that are not needed now, but may be required later, is sequestered but the sequestration is reversible with the correct signal. Those genes that never need to be accessed can be sequestered permanently in much the same manner as one X chromosome is inactivated in mammalian females.

We can now formulate a conceptual model of how all regulons of genes can be divided into one of five categories in any given cell:

1. *Housekeeping Regulons*: regulons always exposed in all cell types.
2. *Differon Regulons*: regulons exposed in a specific cell type, (and by that definition includes the housekeeping regulons).
3. *Differoff - Past Regulons*: regulons that were used in the cell lineage leading up to a given cell type, but have become sequestered in that cell type and cannot be accessed again.
4. *Differoff - Future Regulons*: regulons that are sequestered in a given cell type, but which will become exposed when the cell or its descendants differentiate into new types of cells.
5. *Differoff - Unused Regulons*: regulons that are sequestered in a given cell type and have not been used, are not being used now, and will never be used, by that cell or any of its developmental descendants.

Imagine a system where there are proteins and RNA that use the control elements that exist in the DNA sequence of genes in order to up regulate and down regulate genes. There are also specific signal sequences to sequester or expose whole segments of the DNA. All of this is organized into a hierarchy. The hierarchy was created over evolutionary time as we moved from simple to more complex organisms. During development we can imagine a fertilized egg cell with the whole genome at the beginning of the process. Most of the DNA is reversibly sequestered and not in use. There is an initial exposed section that is regulated by proteins and RNA from the maternal (and to a lesser extent possibly paternal) preloading of the egg. At each signal to differentiate (see next section), the regulating proteins and RNAs respond by exposing one section of DNA and resequestering another section and then chromatin remodeling proteins use epigenetic control such as methylation to seal the sequestration once a section is no longer in use. Since most of the genes will never be used in any one tissue, most of the DNA that is sequestered is never fully exposed. Only the sequestration pattern may change from *Differoff - Future Regulons* to *Differoff - Unused Regulons.*

In more complex organisms, like us, there can be hundreds to thousands of regulons being used, creating hundreds to thousands of different cell types. There are plenty of potential differons: on the order of 2^*N*^ for *N* regulons, though generally only a small fraction of these would suffice and be actually used by an organism. In the determination process, each cell acquires a specific differon (Fig. 4). Changing differons is what underlies and defines embryogenesis. Most organisms have cells that are all totipotent, meaning each cell has the same DNA, but uses determination to select which differon to access in each cell. The main problem is then: how does determination occur, when considered as the selection of which cell uses which differon of genes?

During preparation of germ cells, the entire hierarchical system of DNA organization must be reset. The epigenetic markers of permanent sequestration are removed with each round of meiosis so that *Differoff - Unused Regulons* becomes *Differoff - Future Regulons* (except for a few markers used for keeping track of the paternal versus maternal origin or for DNA repair.) We can now imagine how the genome is used during embryogenesis. The only missing piece is what is the actual signal that tells each cell what part of the genome to sequester and to what level, and what part of the genome to leave exposed. Why is that signal confined to a particular subset of cells? Why is the signal received only at certain times during embryogenesis?

Regulatory gene networks (Fig. 1) are frequently invoked as explanatory for differentiation. A regulatory gene network corresponds to a regulon by our model. If all gene-gene interactions in a particular cell are known and catalogued as a network they would represent a differon in our model. Regulatory gene network models, like gradient models, lack any specific mechanism for defining the timing and the location of specific expression. We think we have found the missing signal. It is differentiation waves transmitted by the cell state splitter organelle.

## The cell state splitter

**Fig. 5:**
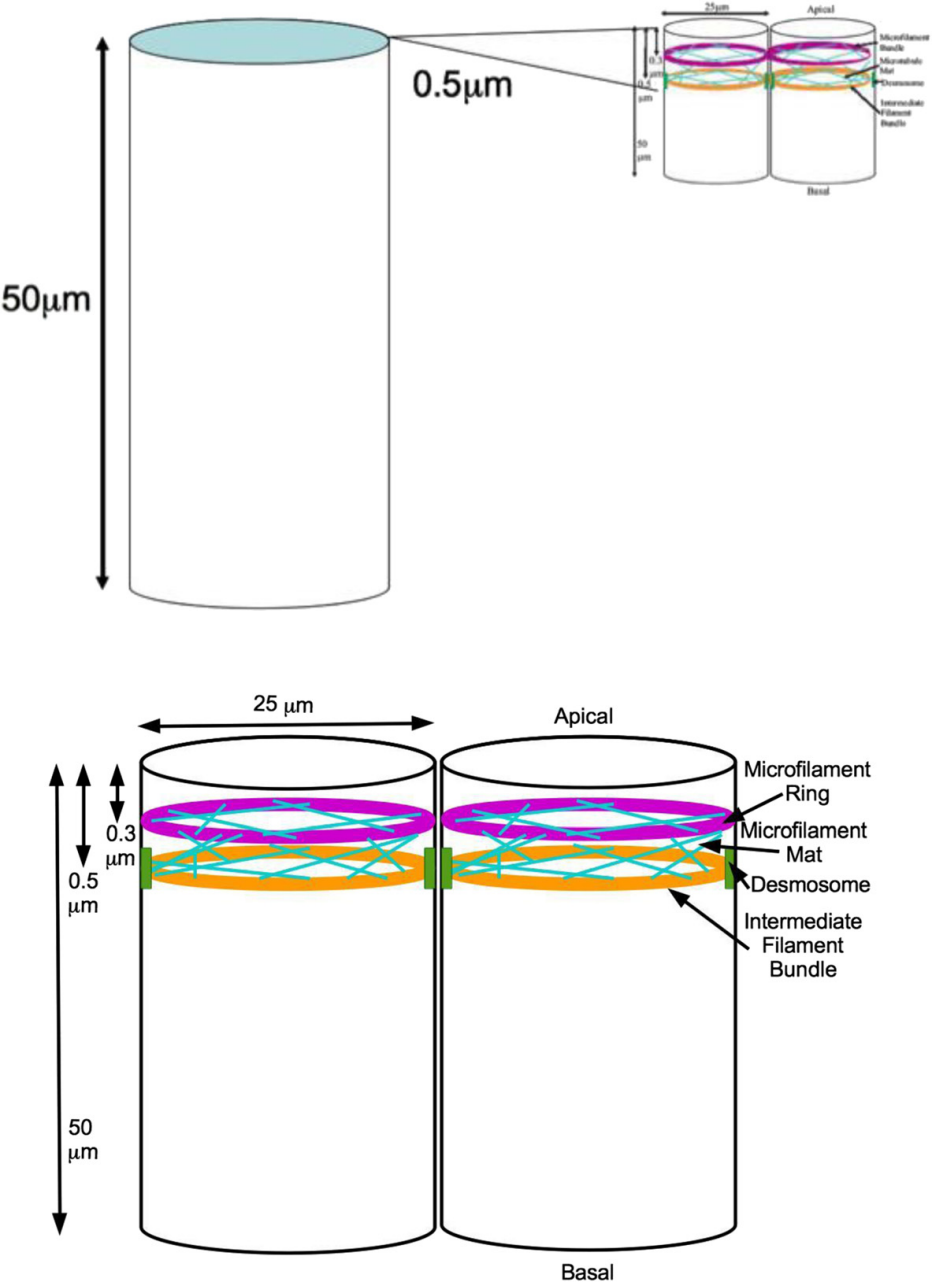
The cell state splitter is an organelle predicted [9] and then at first observed at the apical end of ectoderm cells in axolotl embryos at early gastrulation [11]. It consists of an upper microfilament ring with an intermediate filament ring below it, subtended by a mat of microtubules. As shown in the thumbnail in the upper right, the cell state splitter occupies only 1 % of the height of an ectoderm cell. (Below and in the thumbnail, the vertical and horizontal scales are compressed, as 50 μm = 100 × 0.5 μm)

We first discovered the cell state splitter in the ectoderm of the axolotl [9]. It has the three components: a microfilament ring [122], such as is common in epithelia [123]; a mat of microtubules at and parallel to the apical surface; and an intermediate filament ring (Fig. 5). All three can be seen in electron micrographs of the apical ends of neuroepithelium and epidermal cells. As in all epithelia, the cells in the sheet are also connected at their apical ends by rivet-like structures near the apical end (called tight junctions and adherens junctions). The mat of microtubules may have first been observed by Beth Burnside in the California newt neuroepithelium and epidermis [122, 124], when she was a graduate student with Antone G. Jacobson. Using transmission electron microscopy in an attempt to repeat and confirm Burnside’s work, Chris Martin discovered that the cell state splitter includes the intermediate filament ring and he also showed that the microtubule mat is predominately along the periphery of the cell between the microfilament and intermediate filament rings [11], both at Stage 11 and Stage 15 in ectoderm cells before and after they become epidermal or neural plate cells. Mats of microtubules at the apical ends of cells, ringed by microfilaments, are found in various epithelia Models for apical constriction of epithelial cells are adding details to the mechanism.

The microfilament ring in the cell state splitter does not remain the same thickness as it contracts. Instead the actin ring thickens (cf. [125]). We can infer then that it is not disassembled but instead becomes ever more tightly overlapping in a thickening and strengthening actin cable formation driven [126] by myosin 2, as we proposed for the cell state splitter [9]. Because the actin ring thickens this way, it is able to exert an increasing amount of force as it contracts Conversely, the force will decrease if the cell’s top (apical) diameter becomes larger and the ring is thinned.

## Differentiation waves

The actin ring force is always directed inward, the contraction narrowing the apical end of the cell. Contraction of the microfilament ring can be triggered in three ways. Release of tension across the ring can result in contraction. A sharp abrupt tug on the ring can cause contraction by inducing a smooth muscle type stretch activated contraction response [127, 128]. Once the contraction is underway, if there are connections between cells, biochemical signals relating to the smooth muscle contraction response can cross the gap junctions triggering a contraction in the next cell, which also begins contracting. This then propagates to the next cell, and the next and so on. We now have a wave of contraction propagating across the epithelial sheet of cells. Individual cells will participate in the contraction and then return to their original size. This contraction is a transient event lasting about 10 min per cell in the axolotl. Cells which undergo transformation to bottle cells contract even further but also take about ten minutes per cell. We presume the counterforce to the contraction which returns the cell to their original shape is the intermediate filament ring, acting like a circular spring that can be compressed or stretched and then returns to its resting diameter.

Working in the opposite radial direction from the microfilament ring is the microtubule mat. This set of microtubules goes across the microfilament ring at the edges. They also reach out and downwards to the lower intermediate filament ring (Fig. 5). Altogether they form a circular, annular mat which is open in the middle. The sum of the forces exerted by these individual microtubules, by sliding via motor molecules or polymerization, should result in a force that pushes outward. We anticipate that the force that microtubules exert is approximately constant versus apical radius [9]. Once the expansion due to microtubules occurs in one cell, a signal to expand propagates from cell to cell across a sheet in the same way that a contraction signal can propagate across a sheet. However, unlike with the contraction wave, the expanded cell remains expanded. We presume the slow steady pressure of the microtubules thins both the microfilament ring and alters the intermediate filament ring. The cell becomes flatter and squatter and it stays that way.

Mechanical stresses are now well known to change gene expression. The endothelial cells lining our veins and arteries are subjected to an assortment of powerful mechanical stresses including laminar shear stress, turbulent sheer stress, and circumferential stretch. Each stress activates a specific regulon [129–131]. Most of our knowledge of mechanotransduction comes from studying diseases of the cardiovascular system because the forces there are so obvious and cardiovascular disease is such an important cause of morbidity and mortality. Mechanoreceptors are often surface molecules partially or entirely embedded in the membrane that can sense and respond to mechanical forces. These mechanoreceptors transduce those forces into biochemical signals which are then sent along signal transduction pathways.

These mechanochemical signals are much harder to study because it is difficult to precisely replicate something like shear force. Multiple signals can be received by a cell, and all of these signals can be transduced to biochemical ones, and many will change gene expression. However, the signal transduction pathway typically activated by mechanical stresses is far less specific and less isolated than a purely chemical signal. With mechanochemical signal transduction there is a lot more cross talk and feedback between signal transduction pathways. This makes characterizing mechanochemical signal transduction much more challenging and our understanding has lagged behind that of purely chemical signaling [132].

All of the signal transduction pathways, whether biochemical or mechanical or some combination of both, eventually converge to the level of the DNA. The signal eventually activates or deactivates a specific set of proteins which bind to the DNA at specific regulatory sequences. All of this leads to the transcriptional activation or repression of specific regulons.

**Fig. 6:**
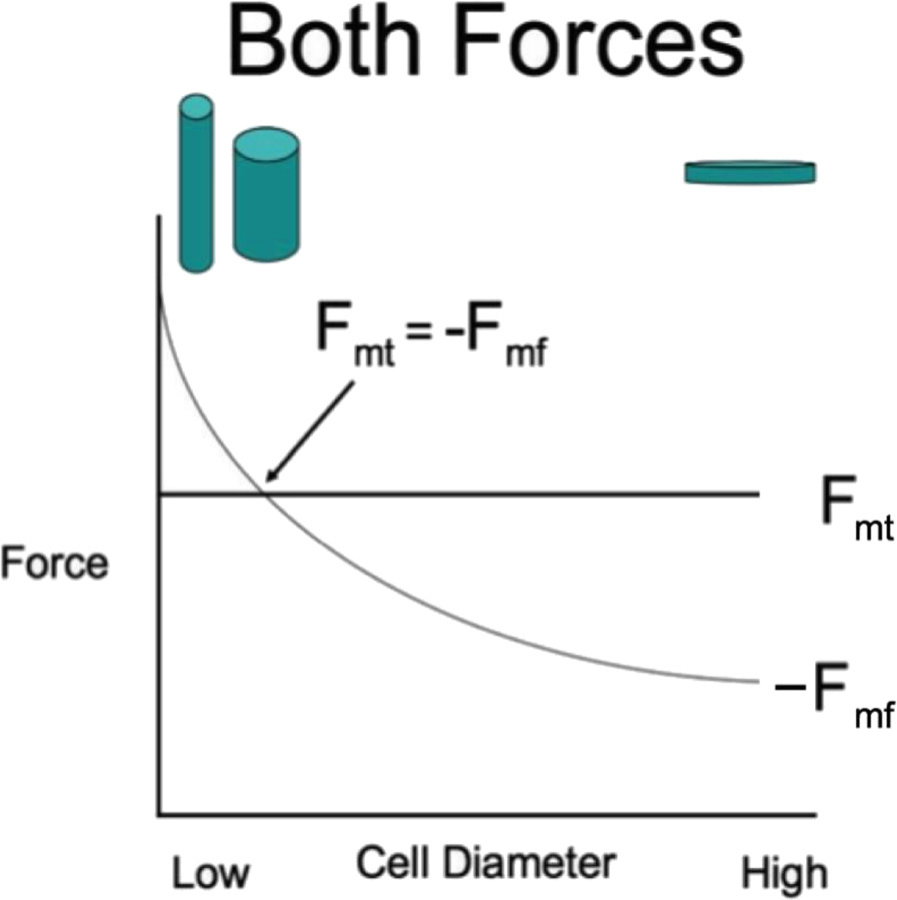
In a cell state splitter, since the outward force due to the apical mat of microtubules (mt) is approximately independent of cell diameter, and the inward acting microfilament ring (mf) force falls of hyperbolically with cell diameter, there should be a cell diameter at which they are in mechanical balance, i.e., *F*_*mt*_ = −*F*_*mf*_. We assume that the cell state splitter is set up at this equilibrium point. However, this is an unstable equilibrium. At higher diameter *F*_*mt*_ > −*F*_*mf*_ and at lower diameter *F*_*mt*_ < −*F*_*mf*_. Thus, if the diameter goes higher than the equilibrium diameter, it will keep getting bigger, flattening the cell. If the diameter goes lower than the equilibrium diameter, it will keep getting smaller, turning the cell into a tall, narrow one. Adapted from Fig. 10 in [9]

We propose that during early development the cell state splitter works entirely on juxtacrine based mechanochemical signaling. Juxtacrine signaling is signaling that requires physical contact and starts with mechanical signals which are then changed to chemical for signal transduction. The result is either contraction or expansion of the apical surface of the cell. We can plot the predicted force balance equation of the microtubule mat versus the microfilament ring (Fig. 6). When a sheet of cells is ready to differentiate, we hypothesize that each has a cell state splitter where the microtubule mat and the microfilament ring are in a bistable balance. This means it is in a configuration that is somewhat stable but is ready to be kicked over into one configuration or another. Each of the cells in the sheet waits for a signal. The signal is an external mechanical signal to tip the balance one way or the other. Once one cell (or a group of cells) within a sheet of epithelia gets that signal, they pass it along to all the other cells in the sheet via an expansion or contraction wave.

If an external force pulls the first cell (or cells) outward in such a way that the microtubules win the tug-of-war, the top of the cell gets wider and wider as the microtubule mat expands and the microfilament ring gets thinner and weaker. Once the microfilament ring has thinned to a certain point there is no turning back. The cell top continues to get wider and wider. The cell participates in a wave of expansion.

If the top of the cell gets narrower due to some external force, the microfilament ring thickens and the top becomes smaller and smaller. Once a certain degree of inward force is achieved, the microtubule mat is completely overwhelmed and the cell cannot get wider again. Microtubule assembly is sensitive to applied forces [133]. If the microfilament contracts with more strength than the microtubule mat can counter, the microtubules will depolymerize.

The intermediate filament ring provides the metastability aspect of the bistable cell state splitter. The intermediate filament ring prevents any minor random fluctuation from triggering the cell state splitter. The force must be large enough to cause a substantial perturbation and shift the balance strongly in favor of either the microfilament ring or the microtubule mat. The intermediate filament ring ensures the specific signal is the trigger and other lesser signals, miscellaneous fluctuations in the mechanical forces, are ignored. This may be represented abstractly by a potential diagram of the metastability. A change in the cytoskeleton with either rapid microfilament ring contraction or rapid microtubule mat expansion is more generally known as a cytoskeletal rearrangement. The activity of the cell state splitter is a specific form of cytoskeletal rearrangement required for determination of cells.

The cell state splitter may be regarded as a tensegrity structure, but as an unusual nonlinear one (Proposition 57 and §3.06 in [5], cf. [134–136]), in that it “snaps through” to one configuration or another from its metastable state. This contributes to the new field of biotensegrity [137, 138].

The standard way of looking at signal transduction from the apical end of the cell stimulation of the canonical Wnt pathway leads to changes in gene expression via β-catenin pathways and it also causes microtubule stabilization for no particular reason. The noncanonical Wnt pathway can lead to microtubule stabilization and to microfilament polymerization and JNK related changes in gene expression. The Wnt-Ca^+2^ pathway leads to microfilament polymerization and calcium related changes in gene transcription. There is no particular reason for the microfilament polymerization to happen either. The changes in the cytoskeleton are just a kind of a peculiar side epiphenomena of no import.

**Fig. 7:**
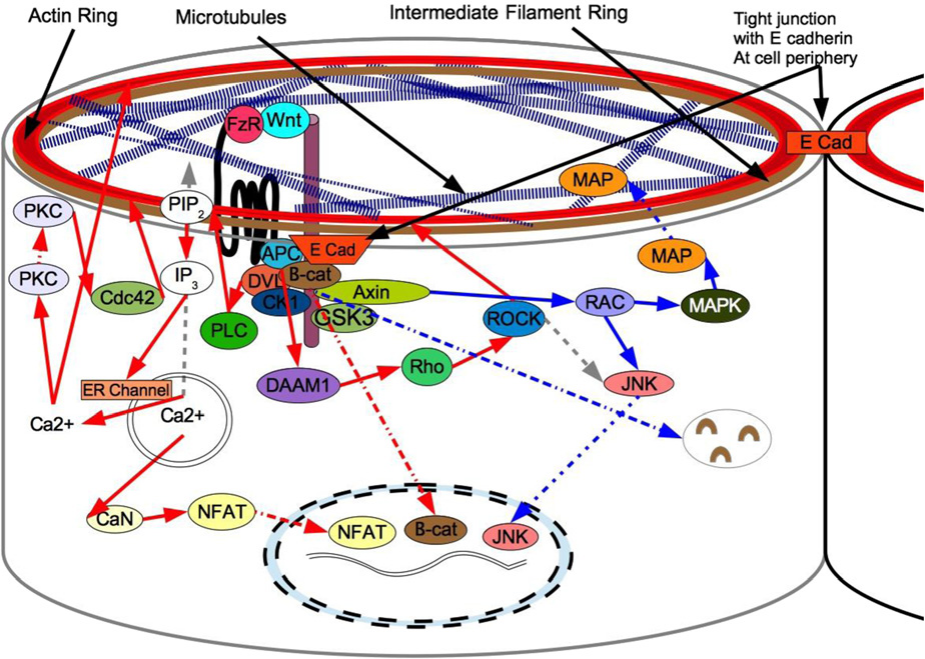
This signal transduction model is general. There will be replacement or alternate proteins for some of the proteins in our model in some tissues or organisms. As embryogenesis proceeds there will be more feedback loops amplifying the contraction and expansion signals and additional inhibitory or excitatory interactions down the signal transduction pathways. In some cellular differentiations, highly specialized versions of these signal transduction pathways will exist such as where cells only one cell state splitter reaction can actually take place. Nuclear State Splitter Participants: ***wnt***- Name is derived from “Wingless” and formerly considered “The Morphogen”, wnt acts primarily as an autocrine protein which binds one or more transmembrane protein and affects their conformation and phosphorylation and thereby affects signal transduction pathways, often amplifying other signals. ***FzR-Frizzled*** transmembrane protein. ***Cdc42*** –a cell cycle protein that takes part in signal transduction pathways and can signal the cell to enter mitosis. It is a member of the small Rho-kinase family that is known to affect microfilament contraction and also can stimulate pathways promoting cell division. A round of determination is often followed by cell proliferation as part of differentiation and this could be via Rho-kinase. ***PKC*** – Protein Kinase C is a protein normally found in the cytosol of the cell in an inactive form but when phosphorylated by other signaling molecules, in particular calcium ions and diaglycerols, translocates to the cell membrane where it interacts with other kinases, especially RACK (receptor for activated C Kinase). PKC continues to signal long after the calcium flux has faded and the diaglycerol signal has ended and so can be considered a signal amplifier. ***CaN***- Calcineurin which is known to dephosphorylate the transcription factor **NFAT**. This causes a conformation change which exposes the NFAT nuclear import signal allowing it move to the nucleus and bind to specific DNA sequences and change gene expression. ***β-catenin*** -Caderin associated protein beta one, a dual function protein that is active in cell to cell adhesion as well as gene expression. *JNK-c*-Jun terminal kinase first found because they bind and phosphorylate c-jun forming a transcriptional activator domain on DNA. **JNK** is a form of mitogen activated protein. ***PLC***-An enzyme which cleaves PIP2 to form two products, inositol 1,4,5-triphosphate (IP3) and diaglycerol both of which are second messengers that commonly affect opening and closing of membrane channels. Diaglycerol and calcium combined can trigger Protein Kinase C translocation. ***PIP2*** -Phosphatidyinositol 4,5-biphosphate, a phospholipid that is the major constituent of membranes that is cleaved by PLC. Such cleavage can activate PKC. ***Dvl*** – Dishevelled, cytoplasmic phosphoprotein that is required for canonical and noncanonical wnt pathway signal transduction and wnt signaling. ***CK1***- Casein Kinase 1 involved in phosphorylation of Dishevelled. Axin- A dual domain cytoplasmic protein, with one domain binding the disheveled receptor and the other binding G proteins. ***APC-***Adenomatous polyposis coli, so called because when one version is mutated in humans it is connected to development of colon cancer. It is a negative regulator which reduces β-catenin response and is also directly connected to e-cadherin. ***GSK3***- glycogen synthase kinase 3, phosphorylates β-catenin (thereby signaling for it to be degraded) in response to signaling from Axin. ***DAAM1***-Disheveled-associated activator of morphogenesis 1, a protein associated with microfilament polymerization, possibly by acting as a scaffold protein. It is activated by Rho. ***Rho*** – A member of the Ras homolog gene family, Rho is a kinase that is activated during microfilament contraction by directly stimulating microfilament polymerization undertaken by the formins. Formins recruit free actin monomers which are then used to elongate microfilaments. (They also capture and stabilize free ends of microtubules required for ruffling in forward movements of cells.) ***ROCK*** – Rho associated protein kinase, this protein works downstream to Rho and can either trigger stabilization or destabilization of microfilaments. It can also trigger contraction by activating specific myosins. ***Rac*** - a serine/threonine-protein kinase that interacts with multiple other kinases. ***MAPK*** – Mitogen activated protein type K. MAPs were originally called microtubule associated proteins because when they phosphorylate another set of proteins, MAP, that bind to microtubules. ***MAP***- microtubule associated protein, bind to microtubules and can either stabilize or destabilize microtubules depending on phosphorylation signals from other proteins. When signalled by wnt signal transduction they generally stabilize and elongate microtubules

The cell state splitter model turns this on its head (Fig. 7). If we assume that the microfilament contraction triggers one change in gene expression and microtubule polymerization triggers another, we can connect the Wnt pathway in a new way and create an updated working model of the cell state splitter. We now have Wnt and the associated proteins sitting in the upper membrane ready to send signals from the cell state splitter organelle to the nucleus. The cell state splitter as an organelle is not itself membrane bound, as are mitochondria and chloroplasts. But it is not unique in this regard. The spindle apparatus and centrioles (microtubule organizing centers) are organelles that are also not membrane bound.

For the cell that starts a differentiation wave, we hypothesize that the initial signal could be purely mechanical. Integrin mechanotransduction is the initial signal, either expansion or contraction for propagating the differentiation wave. That expansion or contraction signal is amplified by the Wnt receptor complex embedded within all its associated structures. With microfilament contraction, we have a calcium flux causing gene transcription via NFAT and β-catenin. With microtubule polymerization and cell expansion, we have a JNK related gene transcription with β-catenin being degraded instead of being sent to the nucleus. The Wnt signal transduction complex serves as a feedback modulator for the functioning cell state splitter apparatus. The Wnt complex signaling is changed via the inhibitory affect on GSK3 and on the AXIN/DVN binding. When microfilaments contract the JNK pathway is inhibited by the action of calcium and direct ROCK inhibition of JNK and because GSK3 is not inhibited, the AXIN/DVN binding is changed to activating DAAM1 and PLC. β-catenin is degraded instead of joining JNK in the nucleus. Thus the cell state splitter at the top of the cell sends a signal, contraction or expansion, and that signal transduction is amplified and passed into the nucleus via the Wnt complex. Gene expression is changed in response. A new differon is accessed.

Profound changes have occurred in the cell because it is now accessing a new cell state splitter signaled differon. This results in changes in gene expression and changes in the types of proteins the cell produces. Changes in which membrane proteins are synthesized, for example, will change the properties of the cell membrane. Expression of different receptor proteins change how and what kind of signal the cell can now respond to. The final process of differentiation involves the setting up of a new cell state splitter to await the next mechanical signal. Differons are turned off or on in much the same way, and using the same signal transduction pathways that are used for changing regulons. The difference is the strength and length of the cytoskeletal rearrangement. This differs sharply from the traditional model of the cytoskeleton as nothing more than a support structure for the cell. In our model the cytoskeletal elements on the apical end of cells in a differentiating represents an organelle of differentiation, the cell state splitter, which is responsible for sensing and transmitting mechanical events during development and signaling those events to the nucleus.

Another point that came out of this work was the long standing embryological assumption that cells have a default fate they follow unless “induced” to do otherwise. So ectoderm will form skin, sweat glands and so forth unless induced to form neural tissue. We do not accept the idea of induction as a deviation from a default fate. Rather we see differentiation as a series of bifurcations where a divergence from one cell type to one of two possible descendant cell types occurs. Once the divergence occurs the original tissue ceases to exist. Thus ectoderm vanishes after the ectoderm waves have passed through it leaving neural epithelium in the wake of the contraction wave and epidermal epithelium behind the expansion wave.

Recall the basic anatomical steps of early embryogenesis. Ectoderm starts as a relatively simple primary tissue type. It subdivides into two tissue types. The ectoderm on the top half of the embryo in late gastrulation goes from rather squat cells to the tall narrow cells or neural ectoderm. These tall narrow cells form the somewhat flat neural plate and then the edges rise and seal into the neural tube which sinks down inside the embryo. In the lower half of the ectoderm, the squat cells expand their apical end and become even shorter and flatter and these flattened cells form epithelial ectoderm, some of which cover the neural tube. Epithelial ectoderm goes on to form skin, sweat glands, and all related external tissue types as development proceeds. We originally studied neurulation out of a desire to understand the human birth defect, spina bifida. We discovered a propagating wave of contraction that went through the portion of the ectoderm that becomes neural ectoderm at the time of neural ectoderm determination. We also found a propagating wave of expansion in the epithelial ectoderm.

Nature surprised us. The actual trajectory of what we came to call the ectoderm contraction wave did not match the first simple mechanical model our group came up with to divide a sphere into two hemispheres. That was a very nice model. Without it we would never have gone looking for the ectoderm contraction wave. But it was wrong. The ectoderm contraction wave begins right at the spot where neural induction occurs about 45° above the original Spemann organizer. It travels as an expanding circle until it breaks over the dorsal lip of the blastopore, then the remaining part travels as an arc. The edges of the arc move more quickly than the middle and so it eventually reforms into a circle. That circle then closes down and vanishes at a point. The wave travels well ahead of the invaginating mesoderm and endoderm as it creates the chamber called the archenteron and so cannot be explained away as simply due to underlying tissue movement.

The ectoderm contraction wave follows this weird trajectory apparently because: 1) a portion of it hits a barrier at the dorsal lip of the blastopore (which turns out to be another differentiation wave) and stops propagating; 2) the remaining arc of the wave is distorted by the involution movement of half of the ectoderm from the outside to the inside of the embryo so that its curvature changes from convex to concave, and the wave then self-annihilates by running into itself in a closing circle. Halting in both cases involves waves running into one another. Not surprisingly, a cell can only participate in one wave at a time. If it is already involved in contracting or expanding in one wave, it can’t simultaneously receive and propagate a second wave. This may be thought of in terms of a refractory period. A cell can also only participate in a wave if the cell state splitter organelle is set up and ready to receive and respond to a signal. In classical embryology terms, if the cell state splitter is ready to respond, the cell is competent.

**Fig. 8:**
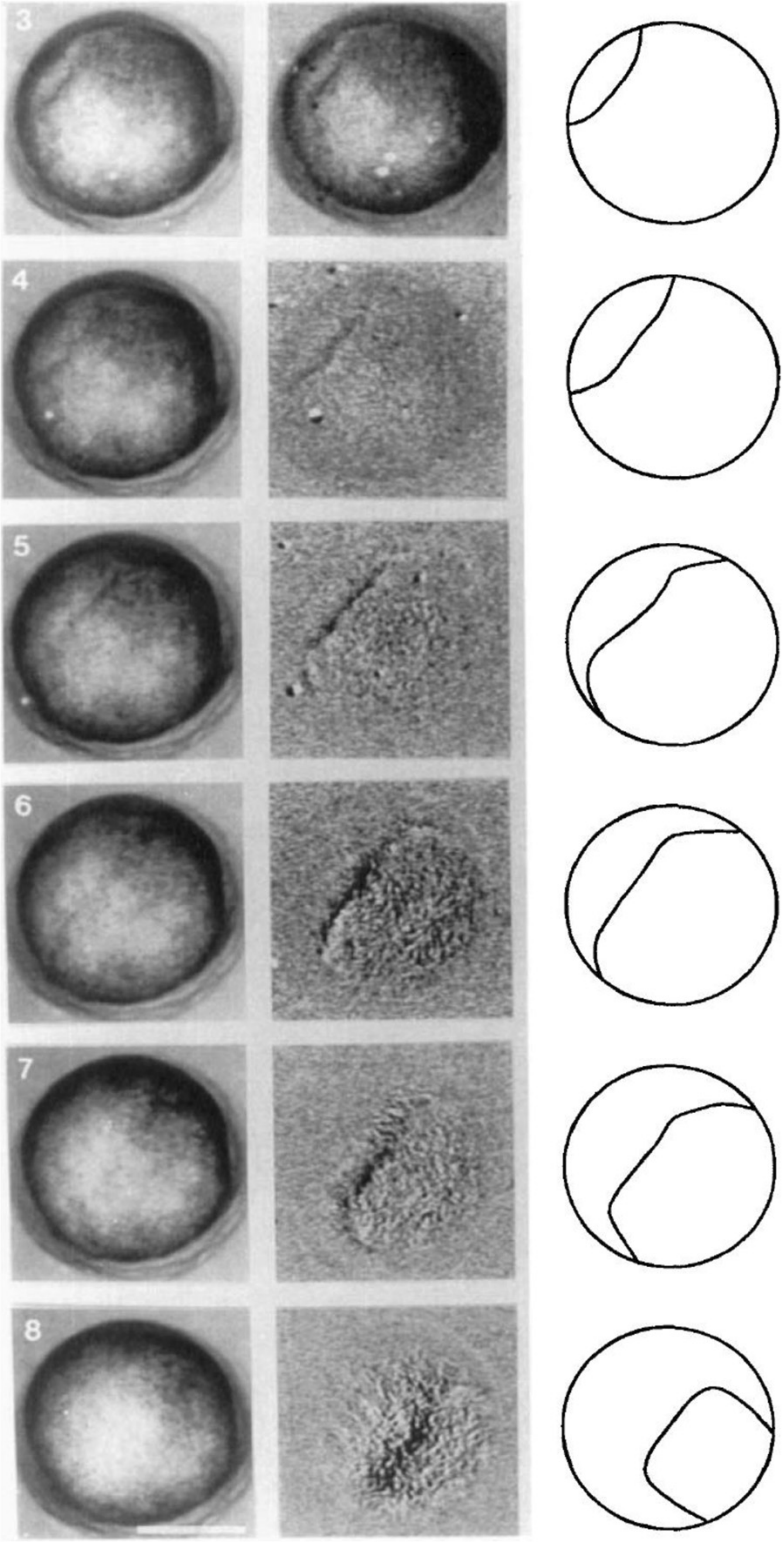
In this axolotl embryo (diameter 2 mm), the ectoderm contraction wave is seen switching from convex to concave as it travels. At its end it closes in on itself and vanishes. From [10] with permission of John Wiley and Sons

**Fig. 9:**
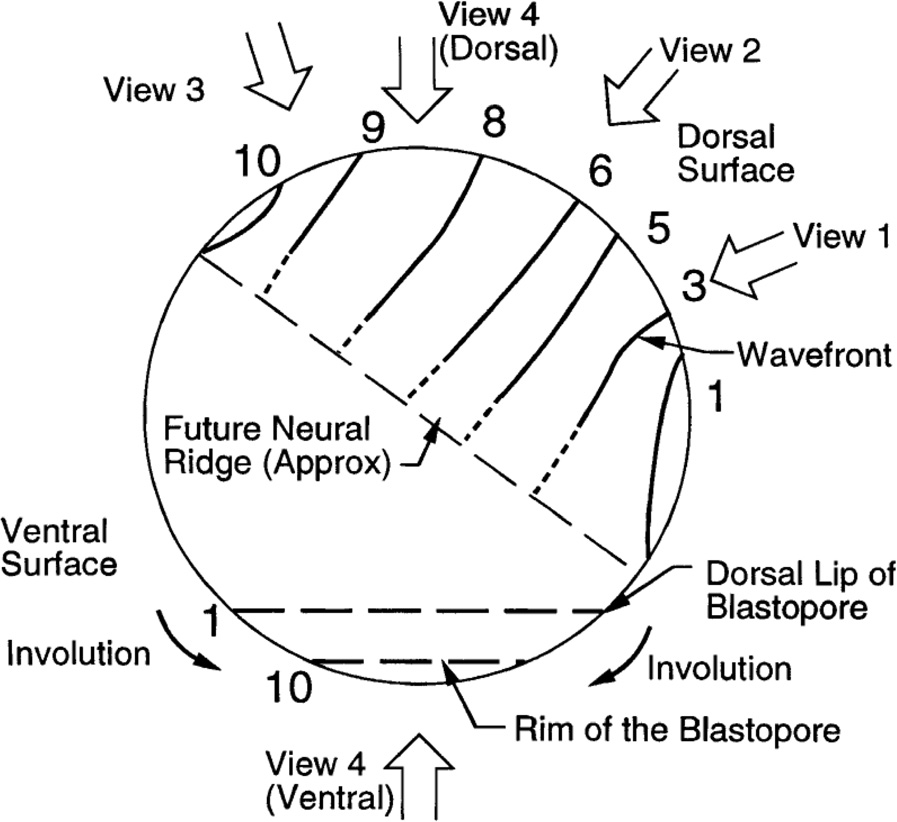
This diagram summarizes how the ectoderm contraction wave moves across an axolotl embryo, as seen from the left side. It starts at a point that spreads to a circle (hour #1), breaking into an arc (hour #3) that moves over one hemisphere, finishing off (hour #10) as a circle that comes down to a point and vanishes. From [10] with permission of John Wiley and Sons. This figure is reproduced and discussed in [183]. This wave is a furrow 0.1 mm wide and deep on this 2 mm diameter embryo propagating around 3 μm/min

We have pictured the ectoderm contraction wave in many ways. This was necessary, because when we started we had no idea how complex its trajectory in time and space would be. First, and most startling, this wave is a deep furrow on the surface of the embryo, made visible with mirrors. We also were initially puzzled that in some embryos the wave spread out or changed from convex to concave (Fig. 8), or shrank to a point and vanished. Using two time-lapse microscopes top and bottom, we discovered that the contraction wave was confined to the upper hemisphere Putting it all together first on a tennis ball, then later by drawing on a clear hamster ball, we reconstructed the trajectory of the wave (Fig. 9). A composite sketch in top view reveals that the wave starts at a point and ends at a point, as if it had gone from one focus to another. The shape is reminiscent of a wave refracting through a gradient index lens.

Let’s now return to the ectoderm cell sheet. Each ectoderm cell has two possible sets of gene expression cascades that are ready to be triggered, i.e., two alternative readied differons. One differon can be triggered by the contraction of the microfilament ring. The other differon can be triggered by an expansion of the microtubule mat. The cell is ready for both events. It is within a cell sheet of similar cells all waiting for a signal. When the external signal comes, the cell either participates in a contraction or an expansion event and a signal is sent to the nucleus. There is an elaborate structure of signaling molecules that connects the cytoskeleton in the cytoplasm to the nucleus. That signaling event is the determination event. The cell nucleus responds by triggering one of the two possible differons. That triggering is determination at the genetic level, and we refer to the process as “nuclear state splitting”. Determination is followed by differentiation, as the genes in the triggered differon are expressed and the resulting RNAs and proteins do their things. The last step in the process is the setting up of a new cell state splitter when it is time for the cells to become determined and to differentiate again. This makes the process iterative, and in a sense recursive. The two choices at each step make the process bifurcating.

This cell sheet differentiation process via cell state splitters may or may not involve cell division (mitosis) in the cell sheet. Commonly, there is a differentiation event, a round of cell division with proliferation and growth of the new cell type, followed by another new round of cell differentiation. These differentiation events and mitosis events often occur in tandem but, unlike single cell division, differentiation and mitosis, or the determination in mosaic embryos, the two do not have to happen together. They are independent. Differentiation can be amitotic.

## Alternate consequences of differentiation waves

After we discovered the ectoderm contraction wave we started looking at the embryo during all stages of development from cleavage through to the end of neural tube closure. We soon found waves occurring all over the embryo at every known major point of determination. We also began finding evidence of similar waves in a host of other organisms. The wide variety of waves we found led us to a general hypothesis of how cells differentiate using differentiation waves.

We can *a priori* expect the following cases of differentiation versus cell division to occur:

- *Proliferative* cell division without differentiation: Both daughter cells are the same type as the mother cell. This is often referred to as a “vegetative”, “proliferative” or “symmetric” [139] cell division.
- *Self-renewing* stem cell division with differentiation: One cell stays of the same type as the mother cell, and the other changes to another kind.
- *Symmetric* cell division with symmetric differentiation: Both daughter cells are of the same kind, but different from the mother cell.
- *Asymmetric* cell division with asymmetric differentiation: Both daughter cells are different from one another, and also different from the mother cell. Empirically we find that one of the pair of daughter cells is generally (but not always [140]) smaller than the other. This is the type of cell division most used by mosaic organisms like the nematode.
- *Amitotic* cell differentiation without cell division: A cell changes cell type without dividing. This is the type we found in the axolotl ectoderm.

The italicized words give us a shorthand for each type. Note that this classification makes no mention of mechanisms or causes of differentiation, nor of where in the embryo the daughter cells end up. In all five cases it is presumed that the amount of DNA in each cell is the same, i.e., these cases do not include meiosis, nuclear division without cell division, or multinucleate cells arising via cell-cell fusion.

We have observed six types of differentiation waves in epithelial cell sheets. Each type has the potential for being either a contraction differentiation wave or an expansion wave. We have observed these waves either in the axolotl during embryogenesis or by careful examination of what is known about determination and differentiation in other organisms. The six types of differentiation waves are as follows. The first three kinds of epithelia generation involve one step of differentiation per cell, while the last three could be called “compound differentiation”, as some cells undergo more than one step of differentiation.

Type 1. *Whole Cell Sheet Differentiation.* The Type 1 form of epithelial differentiation occurs when an entire sheet of cells differentiates together as a coordinated unit forming a new sheet of the next cell type. All the cells in the sheet become the new cell type together. All the cell state splitters do the same thing: they all contract or they all expand. We have observed two forms of the whole cell sheet differentiation waves. There is a moving contraction wave. This begins at one point, then spreads across the whole cell sheet or as a large circle that becomes smaller until it vanishes at a point. The start point is triggered by a mechanical signal generated outside the cells propagating the wave (“external”),  but the propagation from cell to cell within a sheet is internal to the cell sheet. We have also observed contraction where all cells in an entire region simultaneously undergo a contraction or expansion together in response to an external mechanical event. These waves are purely amitotic in nature.
Type 2. *Alternating Patterns of Differentiation.* In Type 2, spatially alternating differentiation is the result. The surface area of the entire sheet remains the same but some cell state splitters contract and some cell state splitters expand resulting in an alternating pattern, often called a spacing pattern [141]. The force driving this alternating pattern has not yet been documented but it is likely that the individual cells in the sheet are relying more on mechanical signals than on biochemical signals. Cell sheets where alternating patterns are found also have far fewer of the structural elements, such as gap junctions, that are required for cell-to-cell communication. Because of the poor cell-cell communication, a wave of contraction or expansion does not propagate from cell to cell. One cell contracts and in doing so, it pulls on the next cell so that the cell expands, which then pushes on the next cell causing it to contract and so on until the entire sheet has experienced alternating contraction or expansion. We have also seen evidence that sometimes it is not a single cell alternating pattern but rather alternating clusters of cells. The result after differentiation is complete is alternating cell types or alternating clusters of cells. This can be a checkerboard pattern of individually different cells or it can produce an alternating pattern of clusters of differentiating cells. If different regions of the sheet start independently of others this will lead to occasional irregularities in the spacing pattern. We have shown that the spacing pattern in *Drosophila* eye imaginal discs that results in its ommatidia begins in a differentiation wave called the “morphogenetic furrow” (Proposition 249 in [5]), but the pattern is also common in spacing of ciliated epidermal cells [142–144], feather buds [145] and plant trichomes [146–148]. We have also observed the alternating patterns of stomata in first leaves and/or cotyledons of seedling *Arabidopsis* plants (cf. [149–157]) This pattern is also amitotic in nature.
Type 3. *Two Cluster Differentiation.* In Type 3 differentiation, there are two clusters of two different kinds of cells eventually formed. The cell state splitters in part of the sheet contract, the cell state splitters in the other part expand. Where expansion or contraction occurs it propagates from cell to cell affecting entire regions of the cell sheet. This is often accompanied by whole cell sheet movement and stress and/or strain caused by the movement, especially where bending occurs. These other mechanical forces limit the propagation of the signal to only one part of the cell sheet. This type occurs during axolotl gastrulation in endoderm, mesoderm, and ectoderm. This type is also amitotic.
Type 4. *Two-Cluster Differentiation with a Few Single Cells*. The Type 4 form of cell sheet is a form of epithelial differentiation which results in two horizontally adjacent cell sheets. This is similar to Type 3 except that Type 4 will also produce a few loose cells. High mechanical stresses at bend points break the gap junctions and cell adherens and this results in some single cells leaving the sheet altogether while the rest of the cells in the sheet remain attached to each other. This typically occurs in regions of bending of the entire cell sheet. The boundary zone, where a few cells are removed, often forms the dividing line between the two new cell types. The cells that stay attached to the sheet differentiate into one of two new cell types depending on whether they have experienced a contraction or expansion (as in Type 3). The detached cells experience neither contraction nor expansion. When individual cells are removed from the cell sheet, the cell state splitter ceases to have any kind of function for them and the cell rounds up instead. The signal for these cells is that they are no longer in the cell sheet. These single cells will, in some situations, take on an amoeboid form and migrate to a new location before differentiating into a new cell type, or they may stay in place and become what are known as pleuripotential stem cells that may become involved in later regeneration. The precise cytoskeletal signal remains to be found but the answer may exist in the study of adherent cells in culture. A cultured cell that is chemically forced to release its adhesion to the culture dish undergoes dramatic cytoskeletal changes and even more dramatic changes to its nucleus. The nucleus changes shape and loses up to 50 % of the volume [158]. These detached cells either migrate to a new location, or they rest near the cell sheet they once belonged to. An example of this type is the neural crest cells of the axolotl. These leave the cell sheet from the boundary zone between neural epithelial cells and future skin cells in the axolotl embryo. These individual ectoderm cells, freed from the cell sheet, give rise to the neural crest cells. Neural crest cells migrate to locations throughout the embryo and eventually form, for instance, the peripheral nerve cells that deliver signals between body and brain, and the pigment cells. This fourth type of wave is also amitotic but cells that fall out of the sheet will continue to have proliferative and/or self renewing divisions once free of the sheet.
Type 5. *Two Cluster Differentiation with Migrating Cells*. Type 5 of cell sheet differentiation is actually a variant of the fourth type. Part of the cell sheet forms two new cell types as in cell sheet Types 2 to 4. The differentiating cell sheet also has cells that leave the cell sheet, often at a point of extreme bending, but instead of just a few cells, many cells will leave the sheet. This new cell sheet can be either beside the old one or they may migrate individually and join together to create a new cell sheet in another location. Type 5-cell sheet differentiation is found in early mammalian gastrulation when the mesodermal layer forms beneath differentiating ectoderm. Mesodermal intercalation in frogs can also be explained as being this form of differentiation. Though mostly amitotic, the cells that produce new migrating cells use other types of cell division such as proliferative, symmetric and asymmetric cell division to accomplish their final result in forming a second sheet of new tissue.
Type 6. *Stratification Differentiation*. Type 6 is a combination of the alternating pattern form Type 2 and the single stem cell form, Type 4, with formation of a new cell sheet below the first. A sheet of cells first differentiates via the alternating pattern of Type 2 producing two new cells types. One of the two new cells forms a new sheet with connections between individual cells and those cells continue to behave as a sheet. The other cell type differentiates to become a stem cell-like cell but that cell remains in place in the sheet. This stem-like cell then begins an asymmetric division in the same plane as the cell sheet plane. This is repeated. The stem-like cells rapidly drop many new cells below the original cell sheet. These new cells, individual products of asymmetric cell division, then proliferate, interconnect and form a second cell sheet layer below the original cell sheet. This form of cell sheet differentiation is important for the thickening and proliferation of the neural tube into the brain once neural tube closure is complete. The place of the stem-like cell within the sheet of other cells keeps it properly oriented. Attachment points act as extrinsic cues allowing the spindle apparatus of individual cells to orient so that the new cell type is always dropped below the sheet [159]. One puzzling aspect of neural cell proliferation is that it is often followed by a round of cell death in an alternating pattern. This pattern of cell death can be explained by all the stem-like cells in the sheet undergoing programmed cell death as their final differentiation once their role of creating the new cell layer below is completed [160]. This round of cell death could be triggered by a subsequent wave through the entire cell sheet.

The action of the cell state splitter is always involved in the determination step. First there is either a microfilament ring contraction or microtubule mat expansion. This creates the determination signal. The signal is either “contraction” or “expansion”. Which signal is actually initiated in a given cell depends on the mechanics and physics of the embryo as a whole. Certain physical areas are mechanically affected such that the microfilament ring wins the tug-of-war and a contraction occurs at one region of cells. Alternatively, the microtubule mat is given the advantage by some mechanical event and the microtubule mat wins the tug-of-war and an expansion wave propagates over one region in the cell sheet.

Embryologists have divided embryos into two types: the regulating embryo and the mosaic embryo. The regulating embryo is one where up to a certain point, the embryo can be split apart but will go on to form two or more fully function though genetically identical individuals. The mosaic embryo cannot do this and if divided will not develop normally. Now let’s take a step back and look at those mosaic organisms. Most often their cell division is asymmetric and produces one smaller cell and one larger cell. If we assume the division is linked and the smaller cell undergoes the equivalent of a contraction and the larger cell undergoes the equivalent of an expansion, then we have a spindle apparatus that is both a cell state splitter and a mitotic spindle. This is a mitotic single cell differentiation wave, the evolutionary precursor to the amitotic differentiation wave.

We have observed that there is an initial ten-minute contraction [161] during determination in the future nerve cells in axolotl ectoderm. This contraction relaxes and the ectoderm cells return temporarily to their previous shape. When the future nerve cells begin differentiating a permanent shape change into tall thin cells occurs. So apical contraction leads to tall skinny cells in ectoderm that becomes neural cells. Similarly, when the expansion wave goes through the lower half of the ectoderm, that expansion is then followed by a change to short squat cells. However, final shape change depends on subsequent differentiation events and not on the determination wave. We should not expect the final shape of all cell types will always be contraction wave = tall skinny cells and expansion wave = short squat cells.

We now have another explanation for so-called “morphogen” gradients. Once any individual cell has participated in an expansion wave or a contraction wave, determination is complete. Differentiation follows. If the cell sheet is examined as a whole during differentiation, there will be a gradient of new protein products that result from differentiation progressing across the sheet. The highest concentration of new proteins will be in the region where the wave began. The lowest concentration will be in the region where the wave ended. This gradient is merely a temporary epiphenomenon, a byproduct of differentiation wave activity. Once all differentiation is complete, the gradient will vanish and all cells of the same type will have the same level of gene expression over the entire sheet. There is one very good use for gradients of gene expression. Wherever they exist, one can expect to find a differentiation wave.

It is plausible that there are differences between early versus late developmental cell state splitter signal events. The nature of the signaling event between cells either in a cell sheet or between cells in asymmetric division is always a combination of a mechanical and a chemical event. Very early in embryogenesis, the mechanical aspects of the signal are much more important. Entire sections of the embryo contract and expand and enormous shape changes occur. In later embryogenesis, the mechanical aspects of the signal are likely to be less important and the biochemical aspects based on receptors to initiate waves or trigger isolated cell differentiation may predominate. Biochemical signals can travel over large distances inside an organism or past other cell types that are untouched by the signal, via the blood, lymph or extracellular space. The use of hormonal molecules with tissue specific receptors is an example of purely biochemical signaling. Biochemical signaling is also important for coordinating a set of cells that are physically dispersed and not connected to other cells of their type such as some nerve cells and blood cells (other than red blood cells lacking nuclei). Such biochemical signaling nonetheless uses the same cytoskeletal elements that are used for purely mechanical signaling. The receptor is simply an adaptation to the cytoskeleton, an extension of it, allowing the cytoskeleton to respond to a purely biochemical determination signal for initiating differentiation instead of a mechanical one.

## The differentiation tree

**Fig. 10:**
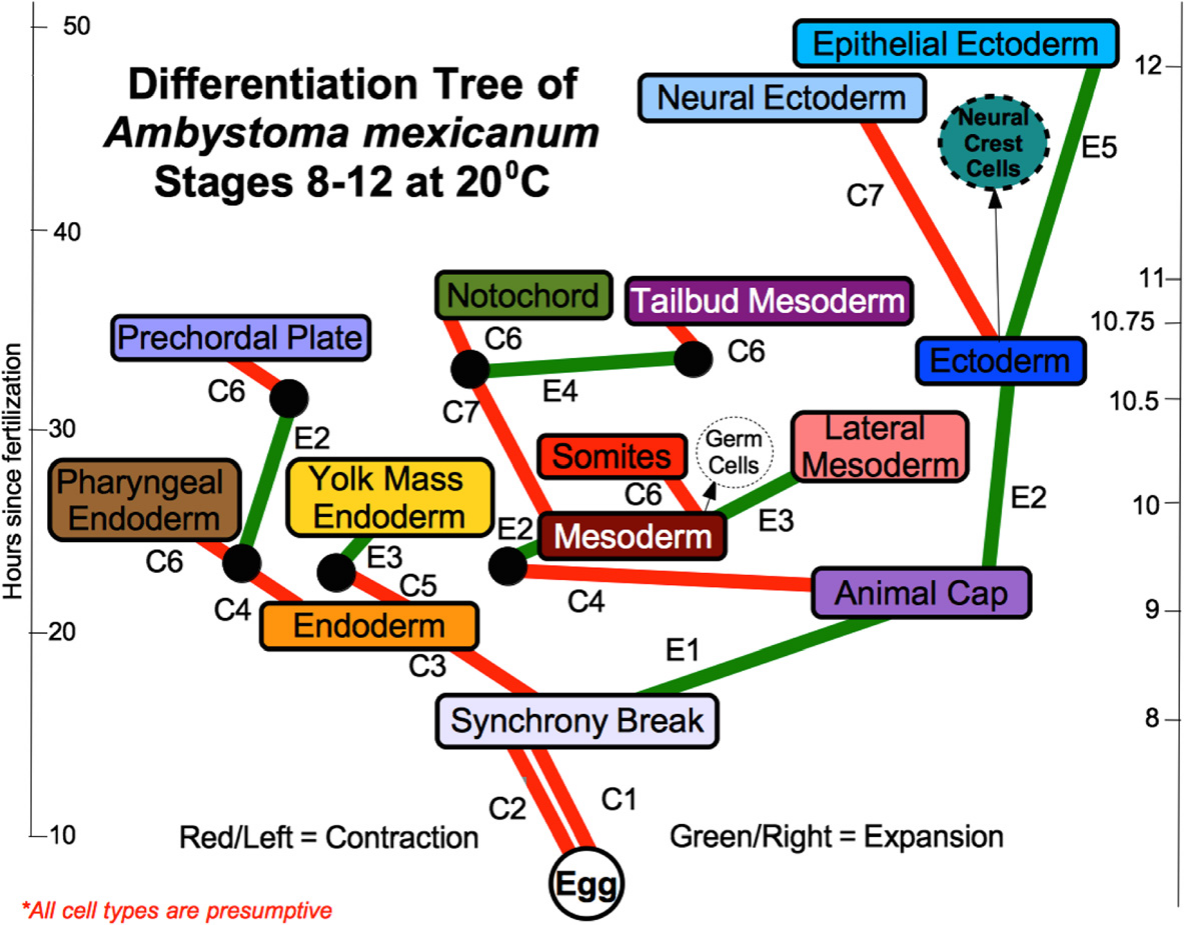
This differentiation tree indicates tissues determined from cleavage to the end of gastrulation. All tissues types should be considered “presumptive”, meaning that their names refer to the kinds of tissues that form from them. Red, shift to right and the letter C indicate a contraction wave and green, shift to right, and the letter E an expansion wave. Staging numbers and hours since fertilization on the left and right are from [184] and indicate the duration of each wave. Note each wave may pass through one tissue and continue into an adjacent tissue and so, for example, E3 begins endodermal tissue and then continues into mesodermal tissue

We end this by introducing the concept of a “differentiation tree” as a way of summarizing what we have so far (Fig. 10). Each tissue in an embryo is designated by a decimal “differentiation code”. The integer to the left of the decimal point is the number of steps of differentiation the tissue has gone through. The binary number to the right of the decimal point shows the sequence of waves that the tissue went through to arrive at its current state: 0 for contraction wave and 1 for expansion wave, with the newest waves to the right in the code. Any horizontal line across the differentiation tree, at a specific developmental time, shows which tissues exist in the embryo at that moment. At each node a tissue splits into two tissues. The portion going through a contraction wave is represented by an arrow tilted to the left, while portion going through an expansion wave is represented by an arrow tilted to the right. Since each tissue is unique, with different subsets of genes exposed and sequestered (different differons), each arrow representing a tissue is colored differently. We regard all of the cells in an embryo that have the same differentiation code as equivalent to one another, in the sense that they have the same subsets of genes exposed and sequestered, i.e., the same differons. Thus to distinguish our notion of a tissue from the less precise uses of the word, we will refer to all cells with the same differentiation code as an “equivalence tissue”. This language conforms to the notion of “equivalence class” in mathematics [162].

If a tissue consists of a single cell, as it usually does in nematodes, for instance, then at each asymmetric division we refer to the larger daughter cell as having undergone a “single-cell expansion wave” and the smaller daughter cell as having undergone a “single-cell contraction wave”. In this case, we may call the organism’s differentiation tree a “cellular differentiation tree”, and the number of cells in any equivalence tissue is 1, i.e., the nematode consists primarily of one-cell tissues, since most of its cells have a distinct differentiation code. We are beginning to compare the differentiation trees of various organisms across widely different phyla [163]. We expect to find some larger unifying structure of differentiation trees, perhaps illuminating the evolution of differentiation.

## Cybernetic embryo

We are now ready to end with our concept of the cybernetic embryo [164]. The idea can be summarized recursively as follows:

1. Each tissue divides into two tissues via a pair of differentiation waves, one contraction wave and one expansion wave.
2. These waves, upon launching, each have a three-part goal: a) spread through a portion of the cells in the tissue, b) cause the cell(s) through which they pass to undergo a step of differentiation, according to the differentiation code and the binary choice (expansion/contraction) of differons (which is encoded in the DNA), c) set up the conditions for launching of the next pair of differentiation waves in the tissue through which the differentiation wave has propagated.
3. For each wave in a pair of waves, go to #1.

The result is a bifurcating cybernetic system, which we represent as a differentiation tree One nuance is that some waves go through more than one tissue, perhaps providing some global coordination [164]. The cell state splitters triggered by these waves, representable by the differentiation code, result in the right kinds of cells in an organism in the right place, at the right time.

## Conclusions

Differentiation waves are reasonably robust, in that they can accommodate variations in cell numbers, cell size, embryo size, and a wide range of temperature and other environmental fluctuations, including the hand of the experimenter, i.e., in multicell tissues they are traversing. By specifying “cell(s)” the above applies equally to mosaic and regulating organisms, and to tissues of both types in both, as most organisms partake of mosaicism or regulation in various degrees, times and places. As a kind of bifurcating relay race, differentiation waves may explain the temperature independence of poikilotherm embryogenesis over a wide range of temperatures, despite the dependence of the overall speed of development on temperature. Perhaps it is the relay race itself that collapses outside the species-specific range of normal development, resulting in gross abnormalities including neural tube defects.

Looked at this way, a regulating embryo is then basically equivalent to a mosaic embryo in terms of its tissues. Pairs of expansion and contraction waves traversing a tissue and dividing it into two new tissues then are but extensions of the asymmetric cell division. We’ve then come full circle. Instead of regarding an asymmetric cell division as a pair of single-cell differentiation waves we regard an “asymmetric tissue division” via a pair of expansion and contraction waves as if it were a big “tissue-cell” undergoing an asymmetric “tissue-cell” division. If the notion works [163, 165], it will unify concepts of development across a vast array of phyla. Perhaps we and the worm are indeed but one in developmental mechanism: there is some degree of universality to differentiation trees, and this is why our numbers of genes are similar.

With our discovery of differentiation waves, the cybernetic embryo has become a testable hypothesis. As is required of any good theory [166], we can observe and measure and decide if each differentiation wave meets criteria for a goal directed system, and what those goals are. After all, if you walked into a room and saw a device on the wall, you could play with it, open the window, trace its wiring (or wireless) communication means with furnaces, air conditioners, automatic window openers, etc., and determine its goals and robustness in meeting those goals. We don’t usually think of thermostats this way, but they can be reverse engineered, and so can the embryo [167–181]. By thinking of the embryo in terms of differentiation waves that have goals in the cybernetic sense, we have a theory of how the embryo builds itself that is experimentally testable. The model only requires we accept the cell state splitter as the organelle of differentiation.
